# Supplementary material for: Suppression of external quantum efficiency rolloff in organic light emitting diodes by scavenging triplet excitons
Source: Nat Commun. 2020 Oct 1;11:4926. doi: 10.1038/s41467-020-18292-0 (PMC7531006; doi:10.1038/s41467-020-18292-0)
Supplement: Supplementary file 1 — Supplementary Information [file 41467_2020_18292_MOESM1_ESM.docx]

**Supplementary Information**

**Suppression of external quantum efficiency rolloff in organic light emitting diodes by scavenging triplet excitons**

*Buddhika S. B. Karunathilaka, Umamahesh Balijapalli, Chathuranganie A. M. Senevirathne, Seiya Yoshida, Yu Esaki, Kenichi Goushi, Toshinori Matsushima*, Atula S. D. Sandanayaka* and Chihaya Adachi**

**Supplementary Figures**


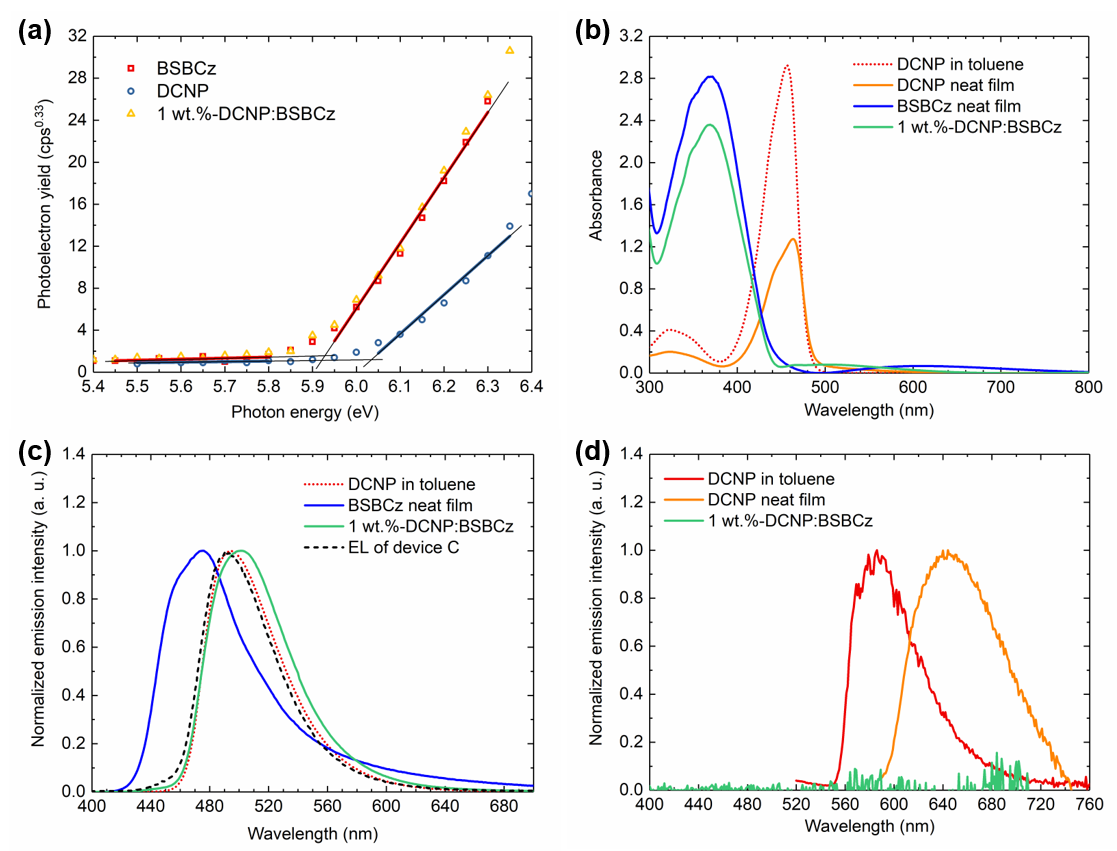


**Supplementary Figure 1. Photophysical parameters of materials.** (a) Photoelectron yield spectra of 100 nm-thick films of neat BSBCz, neat DCNP and 1 wt.%-DCNP:BSBCz. (b) Absorbance spectra of 1×10^-5^ M solution of DCNP in toluene, 200 nm thick films of neat DCNP, neat BSBCz and 1 wt.%-DCNP:BSBCz at ambient temperature. (c) PL spectra of 1×10^-5^ M solution of DCNP in toluene, 200 nm thick films of neat BSBCz and 1 wt.%‑DCNP:BSBCz at ambient temperature and EL spectrum of device C. (d) Phosphorescence spectra of 1×10^-5^ M solution of DCNP in toluene and 200 nm thick films of neat DCNP and 1 wt.%-DCNP:BSBCz at 77 K.


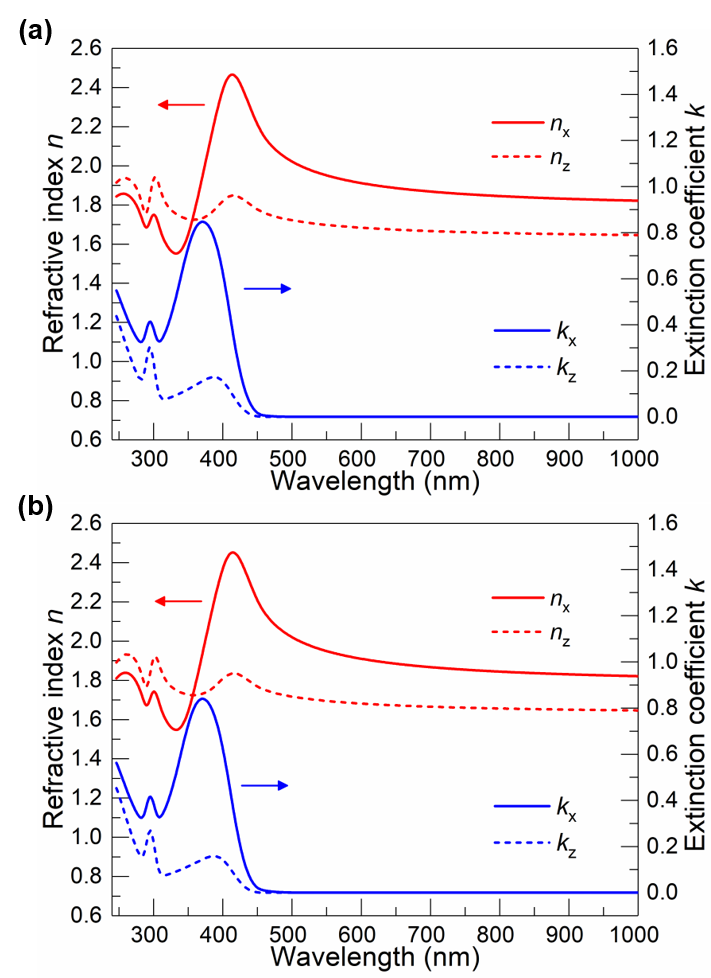


**Supplementary Figure 2.** **Molecular orientation of materials.** Spectra of refractive indices and extinction coefficients of (a) BSBCz neat films (100 nm) and (b) 1 wt.% DCNP:BSBCz films (100 nm), which were obtained using VASE and a uniaxial anisotropic fitting model.


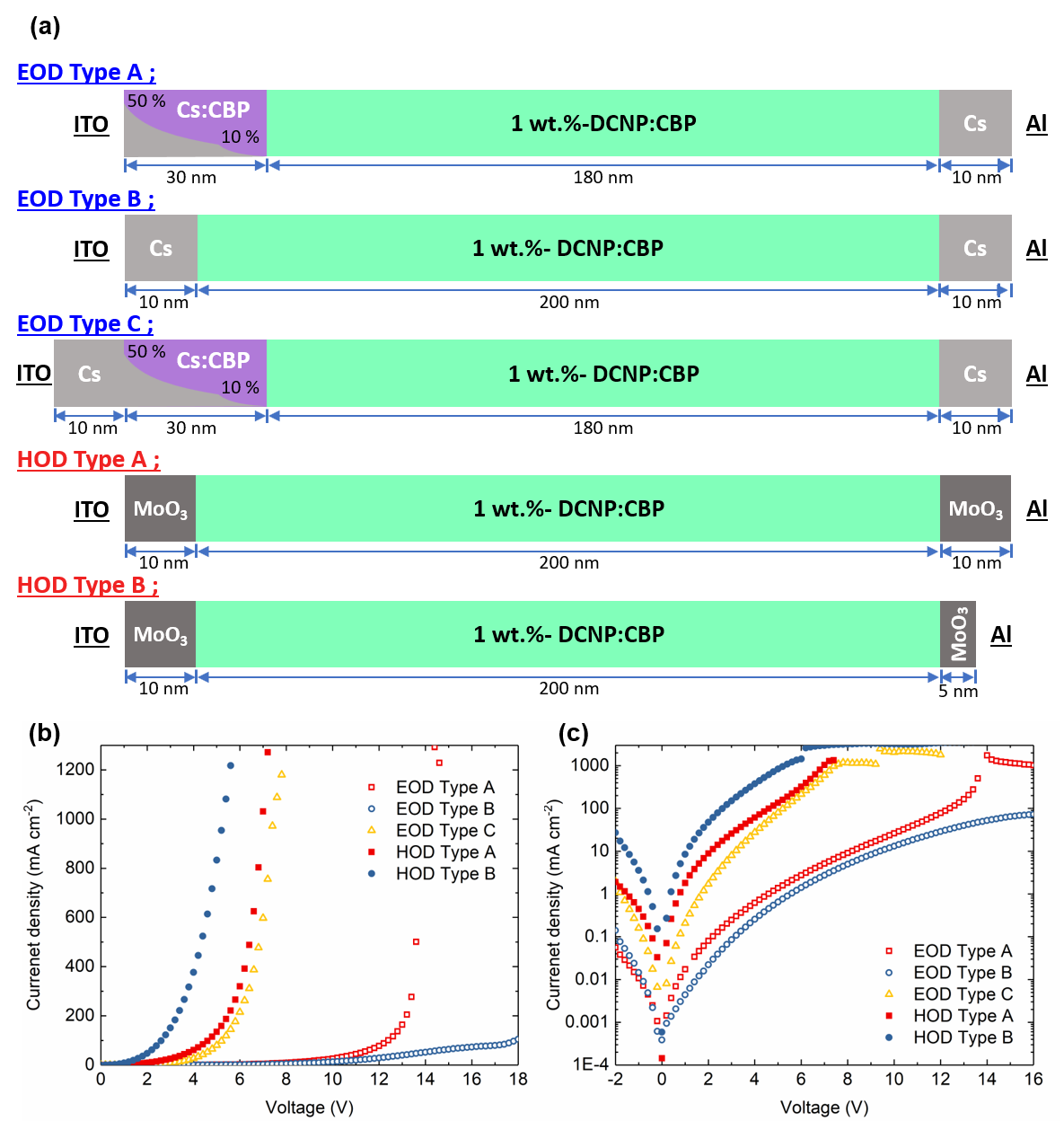


**Supplementary Figure 3.** **Optimization of charge balance in a single-layer OLEDs with CBP.** (a) Device architectures of EODs and HODs with a 1wt.% DCNP:CBP single layer and varying electron injection and hole injection layers. Current density−voltage (J−V) curves of EODs and HODs in (b) linear plot and (c) semi-log plot. From the comparison of EOD type A, B and C, the combination of 10 nm thick-Cs and Cs doped layer enhanced electron injection efficiency, while the decreased MoO_3_ thickness enhanced hole injection efficiency. Based on these results, we found that the combination of EOD type C and HOD type A provides the optimum charge balance specially at high current density under DC operation.





**Supplementary Figure 4.** **Photophysical properties of a C545T laser dye.** Absorption, fluorescence spectra at room temperature and phosphorescence spectra at 77 K for C545T in solutions and solid-state films.


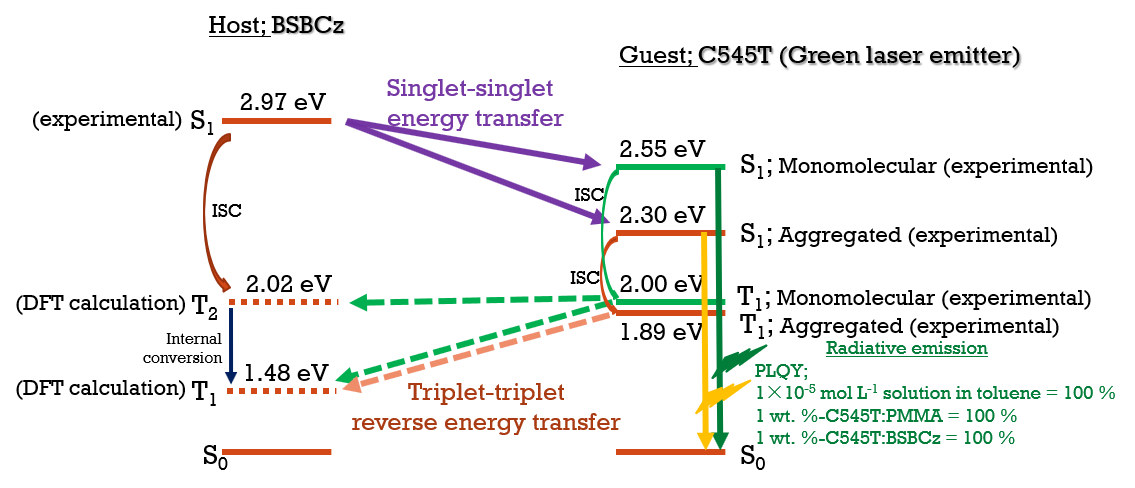


**Supplementary Figure 5. Energy-level diagram of a C545T:BSBCz guest-host matrix.** The energy levels admit that BSBCz can use as a triplet scavenging host material for C545T laser dye.


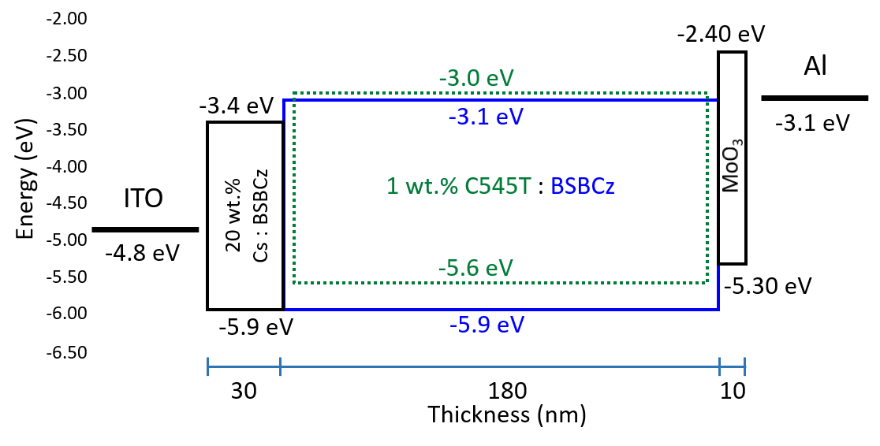


**Supplementary Figure 6.** **Energy-level diagrams for single layer OLEDs having C545T.** Based on HOMO and LUMO energy levels, C545T can use as the emitter while BSBCz use as the host. C545T do not work as strong carrier traps.


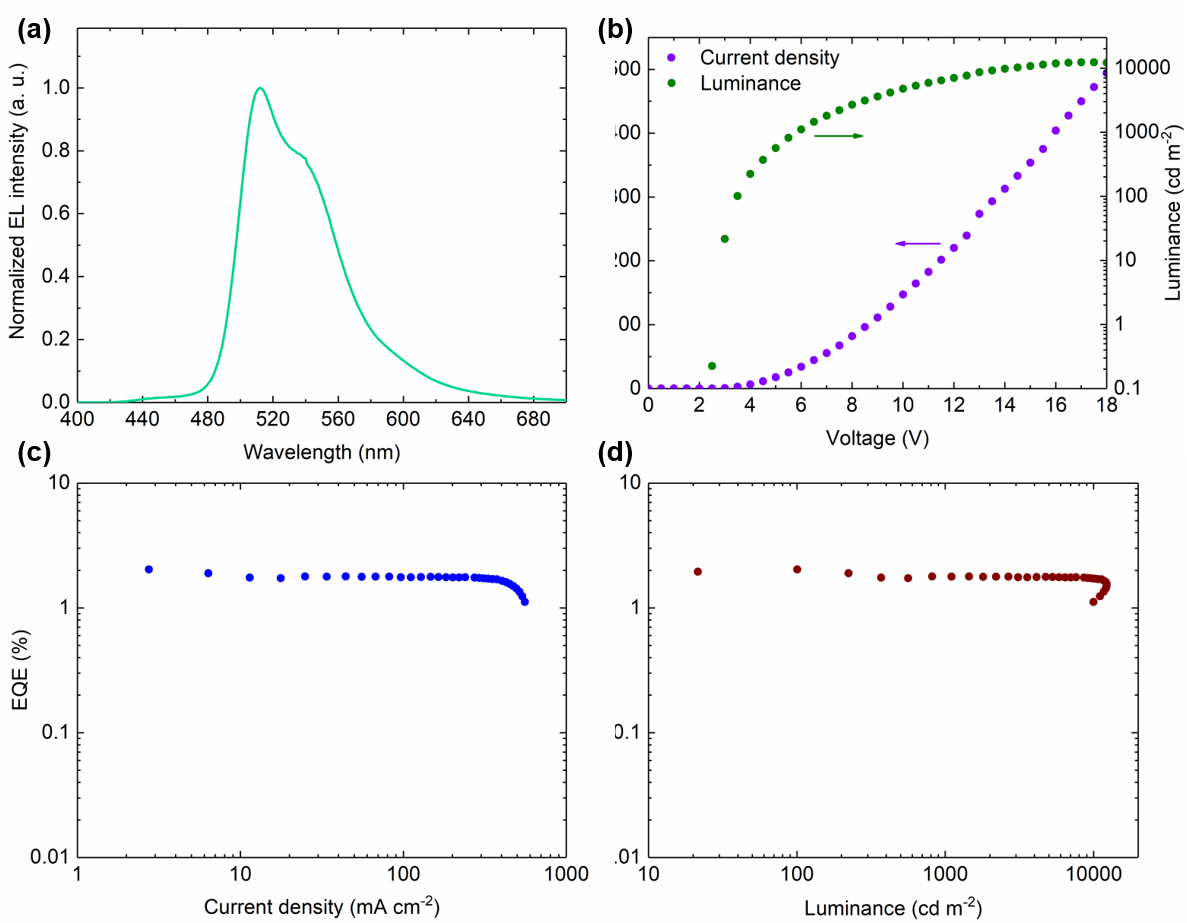


Supplementary Figure 7. Characteristics of OLED with 1 wt.%-C545T:BSBCz as EML. (a) The EL spectrum is similar to the PL of C545T with no extra shoulder peaks, suggesting the efficient FRET from host to guest. (b) Current density−voltage and luminance−voltage curves showing similar results as Device C (c) EQE−current density curve showing no serious EQE rolloff until the device break-down due to joule heating. (e) EQE−luminance curve showing the maximum luminance over 10000 cd m^-2^ with no rolloff of EQE until the device break-down.


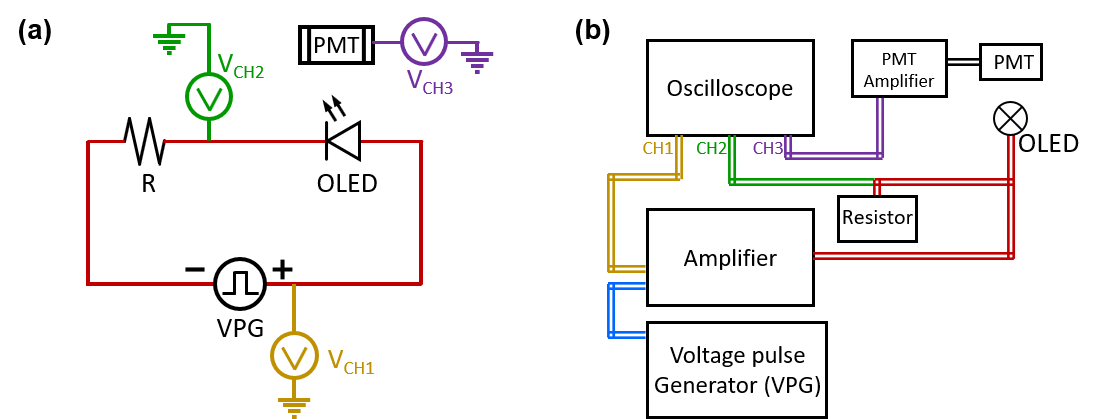


**Supplementary Figure 8.** **Transient EL measurement setup.** (a) Circuit diagram of our transient EL measurement setup using square pulsed voltages. (b) the same setup as a schematic of showing BNC cable connections.


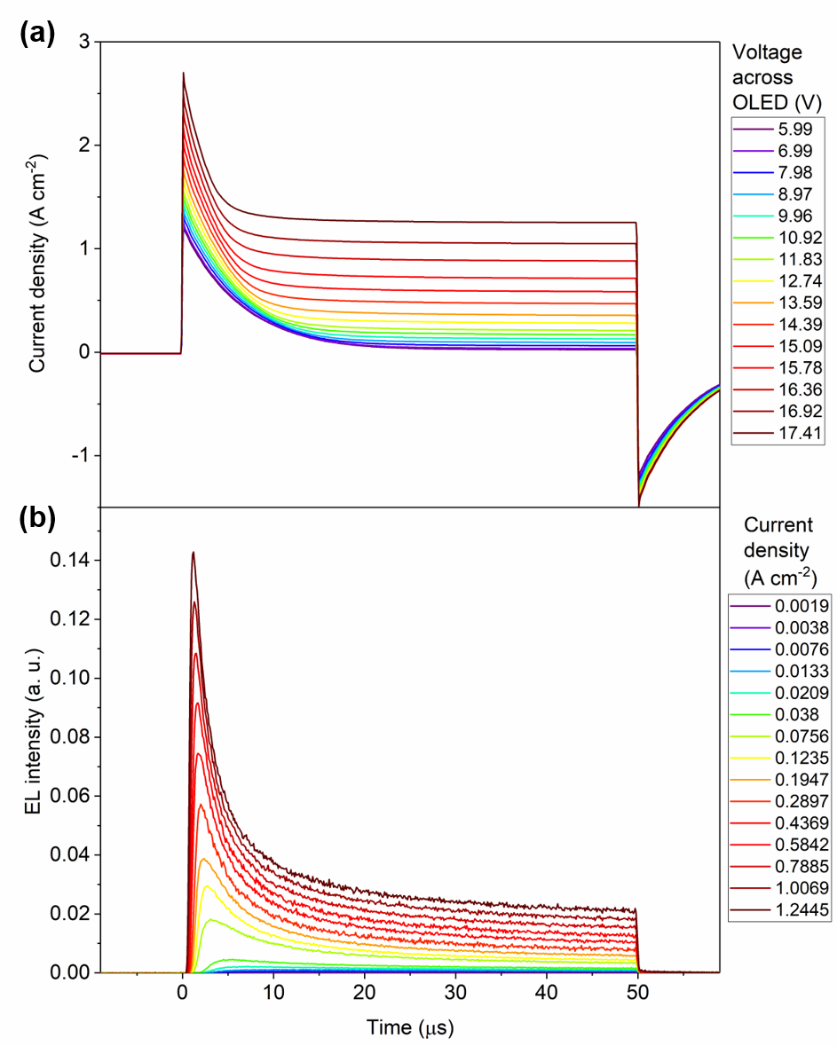


**Supplementary Figure 9. Current density and Transient EL responses for device A.** (a) Current density and (b) EL intensity responses of **device A** under pulse operation with a width of 50 µs. The capacitance current flow was observable from 0 to <10 µs and then OLED driving current flow stayed unchanged while EL response was gradually decreased.


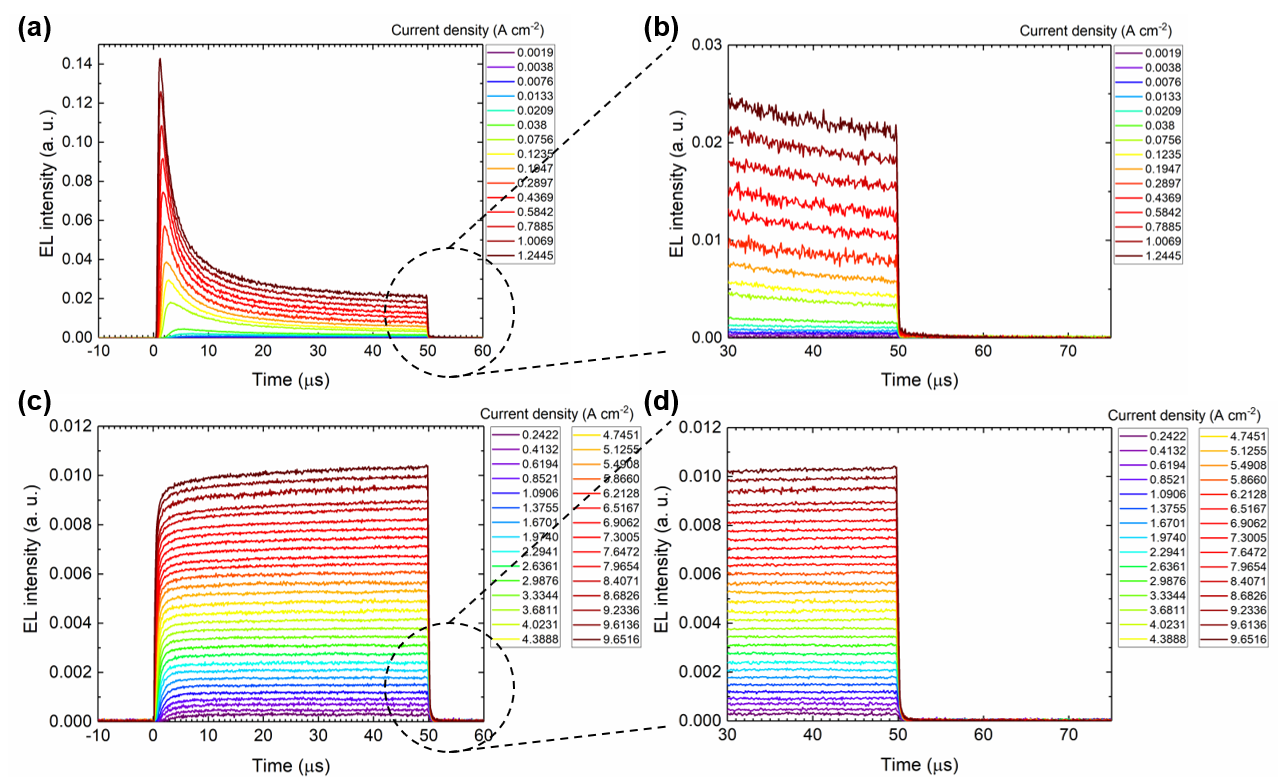


**Supplementary Figure 10. Off-state EL responses of OLEDs.** (a,b) With the host of CBP for device A and (c,d) with the host of BSBCz for device C. Different current densities with a pulse width of 50 µs were applied to these devices. In the expanded figures (b,d), both devices with CBP and BSBCz hosts showed no off-state EL, indicating that no serious charge accumulation occurred in both devices.
